# Vascular Calcification and the Gut and Blood Microbiome in Chronic Kidney Disease Patients on Peritoneal Dialysis: A Pilot Study

**DOI:** 10.3390/biom12070867

**Published:** 2022-06-21

**Authors:** Ana Merino-Ribas, Ricardo Araujo, Luciano Pereira, Joana Campos, Luísa Barreiros, Marcela A. Segundo, Nádia Silva, Carolina F. F. A. Costa, Janete Quelhas-Santos, Fábio Trindade, Inês Falcão-Pires, Ines Alencastre, Ioana Bancu Dumitrescu, Benedita Sampaio-Maia

**Affiliations:** 1 Nephrology & Infectious Diseases R & D Group, i3S—Instituto de Investigação e Inovação em Saúde, INEB—Instituto de Engenharia Biomédica, Universidade do Porto, 4200-135 Porto, Portugal; anamerinoribas@gmail.com (A.M.-R.); ricardo.araujo@i3s.up.pt (R.A.); lucianoarturpereira@hotmail.com (L.P.); joanaaraujocampos@gmail.com (J.C.); carolina.costa@i3s.up.pt (C.F.F.A.C.); ines.alencastre@ineb.up.pt (I.A.); 2 Departament de Medicina, Universitat Autonoma de Barcelona, 08035 Barcelona, Spain; ioana_bancu@yahoo.com; 3 Nephrology Department, Hospital Universitari de Girona Doctor Josep Trueta, 17007 Girona, Spain; 4 Nephrology Department, Centro Hospitalar Universitário de São João, 4200-319 Porto, Portugal; nadiaraquel77@gmail.com; 5 LAQV, REQUIMTE, Departamento de Ciências Químicas, Faculdade de Farmácia, Universidade do Porto, 4050-313 Porto, Portugal; lbarreiros78@gmail.com (L.B.); msegundo@ff.up.pt (M.A.S.); 6 Instituto de Ciências Biomédicas Abel Salazar, Universidade do Porto, 4050-313 Porto, Portugal; 7 UnIC@RISE- Cardiovascular Research and Development Centre, Department of Surgery and Physiology, Faculty of Medicine, University of Porto, 4200-319 Porto, Portugal; sjanete@med.up.pt (J.Q.-S.); ftrindade@med.up.pt (F.T.); ipires@med.up.pt (I.F.-P.); 8 Fresenius Nephrocare, 110372 Pitesti, Romania; 9 Faculdade de Medicina Dentária, Universidade do Porto, 4200-393 Porto, Portugal

**Keywords:** chronic kidney disease, vascular calcification, gut microbiome, blood microbiome, mortality risk, sCD14

## Abstract

Vascular calcification (VC) is a frequent condition in chronic kidney disease (CKD) and a well-established risk factor for the development of cardiovascular disease (CVD). Gut dysbiosis may contribute to CVD and inflammation in CKD patients. Nonetheless, the role of gut and blood microbiomes in CKD-associated VC remains unknown. Therefore, this pilot study aimed to explore the link between gut and blood microbiomes and VC in CKD patients on peritoneal dialysis (CKD-PD). Our results showed relative changes in specific taxa between CKD-PD patients with and without VC, namely *Coprobacter*, *Coprococcus* *3*, *Lactobacillus*, and *Eubacterium eligens* group in the gut, and *Cutibacterium*, *Pajaroellobacter*, *Devosia*, *Hyphomicrobium*, and *Pelomonas* in the blood. An association between VC and all-cause mortality risk in CKD-PD patients was also observed, and patients with higher mortality risk corroborate the changes of *Eubacterium eligen*s in the gut and *Devosia* genus in the blood. Although we did not find differences in uremic toxins, intestinal translocation markers, and inflammatory parameters among CKD-PD patients with and without VC, soluble CD14 (sCD14), a nonspecific marker of monocyte activation, positively correlated with VC severity. Therefore, gut *Eubacterium eligens* group, blood *Devosia*, and circulating sCD14 should be further explored as biomarkers for VC, CVD, and mortality risk in CKD.

## 1. Introduction

Chronic kidney disease (CKD) is a major public health problem carrying a high socio-economic burden with elevated morbidity and mortality [1]. It is expected that CKD will become the fifth global cause of death by 2040 [2]. Cardiovascular disease (CVD) is the leading cause of death among CKD patients with a mortality rate 30 times higher than the general population [3]. The increased CVD risk in CKD patients is only partially explained by traditional cardiovascular risk factors such as diabetes, hypertension, dyslipidaemia, smoking, obesity, among others. Non-traditional risk factors such as inflammation, oxidative stress, endothelial dysfunction, and vascular calcification (VC) have been identified as key players in the development of CVD in these patients [4].

Under normal conditions, inflammation can arise as a protective physiological response to various inimical stimuli. However, in several debilitating disorders, such as CKD, the inflammatory process becomes persistent and contributes to the aggravation of the disease [5]. In CKD, inflammation is likely a consequence of multifactorial aetiology and interacts with several factors that emerge in response to the accumulation of uremic toxins due to renal function impairment, contributing significantly to the higher CVD risk in CKD [6].

A disturbed or unbalanced gut microbiota, described as gut dysbiosis, is currently recognised as a key factor in the pathogenesis or progression of CKD. This CKD dysbiotic ecosystem is characterized by a shift towards proteolytic metabolism mostly due to an increased number of bacteria that possess urease, uricase, and p-cresol, and indole-forming enzymes, and by a decline in saccharolytic fermentation, leading to a reduction of short-chain fatty acids (SCFA) production [7,8,9]. CKD-associated gut dysbiotic state leads to an increase of uremic toxins derived from the microbial metabolism (such as trimethylamine N-oxide (TMAO), p-cresol sulfate (PCS), indoxyl sulfate (INDS), and indole-3-acetic acid (3-IAA) further contributing to the chronic status of oxidative stress and inflammation, and the consequent increase in CVD risk [10,11,12]. Moreover, CKD-related gut dysbiosis is also associated with an impaired epithelial barrier, a condition commonly referred to as leaky gut, which allows the translocation of living bacteria, endotoxins (lipopolysaccharides (LPS), bacterial DNA, and gut-derived uremic toxins into the systemic circulation [13], eliciting or further aggravating the inflammatory state [14]. Together, these data highlight the potential role of gut microbes in CKD and associated CVD.

Beyond the gut, an increasing body of evidence supports the existence of a human blood microbiome with relevance in health and disease, although its origin, structure, and function remain unrevealed [15,16]. Different reports suggested that blood owns a unique microbiome and that a dysbiotic blood microbiome is associated with different pathologies such as atherosclerosis, CVD, ischaemic stroke, and liver fibrosis [17,18,19]. Specifically, in CKD, a recent study showed a blood microbiome profile with lower alpha diversity and significant taxonomic variations when compared with healthy controls [20].

VC and its severity have long been recognized as an important factor in CVD development in CKD patients [21]. VC is an active and highly regulated cellular process defined by the deposition of calcium-phosphate crystals within the intima and media layers of the vasculature and/or heart valves. Several factors have been related with VC, such as biomarkers of inflammation (for example high-sensitivity C-reactive protein (PCR), interleukin (IL)- 6, Tumour necrosis factor-α (TNF-α), and of monocyte activation (for example soluble CD14 and CD163) [22]. In fact, the mineral bone disorder associated with CKD is characterised by one or more abnormalities in circulating minerals and their regulating hormones, bone abnormalities, and VC [21]. Mounting evidence indicates that the gut dysbiosis associated with CKD may be involved in the pathogenesis of bone–vascular axis [8,23]. Recent data suggest that an increased protein fermentation, and consequent uremic toxins production, decreased carbohydrate fermentation, vitamin K deficiency, and gut-derived inflammation may, alone or together, drive to a vascular and skeletal pathobiology in CKD patients [8,23]. Still, to our knowledge, there are currently no data on the putative association between blood microbiome and vascular calcification.

Given the importance of VC in CKD and the associated increased risk of CVD in these patients, the aim of our study was to explore the link between VC, all-cause mortality risk, and the gut and blood microbiome in CKD patients on peritoneal dialysis (CKD-PD).

## 2. Materials and Methods

### 2.1. Study Design, Subjects, and Sample Collection

This cross-sectional observational study included 44 CKD patients undergoing peritoneal dialysis in Centro Hospitalar Universitário de São João in Porto, Portugal, between 2018 and 2019. This study was approved by the local Ethics Committee (approval references 200/18), in accordance with the 1964 Helsinki declaration and its later amendments. All participants were recruited voluntarily after receiving detailed information on the study protocol. Written informed consent was obtained from all patients. Exclusion criteria included age under 18 years old, inability to give informed consent, history of infection in the last 3 months, and antibiotic intake in the last 3 months.

Relevant clinical and demographic information was gathered for each participant. Clinical characteristics collected were gender, age, CKD aetiology, history of high blood pressure, diabetes mellitus, dyslipidaemia, obesity (defined as body mass index of 30 kg/m^2^ and higher), and history of cardiovascular disease (peripheral vascular disease, ischemic cardiomyopathy, or cerebrovascular disease). Their pharmacological treatment and infection history was also gathered.

VC was estimated in all patients using Adragao score through hands and pelvic radiographies [24]. The Charlson Comorbidity Index was also calculated predicting 10-years survival in patients with multiple comorbidities [25,26].

Blood samples were collected in the peritoneal dialysis unit, and the self-collected stool specimens were brought refrigerated by the patient within 48 h after collection. Whole blood and stool samples were collected in DNA-free sterile containers and were immediately frozen and stored at −80 °C for microbiome analysis. Plasma was obtained after blood centrifugation (1500× *g*, 15 min, 4 °C) and stored at −80 °C for biochemical analysis.

### 2.2. Sample Processing and Microbiome Analysis

Genomic DNA was isolated in a strictly controlled environment at Vaiomer SAS (Labège, France) as previously described [20]. Total DNA was extracted from whole blood (100 µL) using a specific Vaiomer protocol carefully designed to minimise any risk of contamination between samples from the experimenters or the environment. Negative controls (molecular grade water added in an empty tube, the same used for sample storage and peritoneal dialysis solution) were extracted, amplified, and sequenced at the same time as the samples. PCR amplification was performed using universal primers targeting the V3-V4 region of the bacterial 16S rRNA gene (340F-781R). Illumina sequencing length, by use of the 2 × 300 paired-end MiSeq kit V3, was designed to encompass the 476-base pair amplicons. Sample multiplexing and sequencing library generation were conducted, as previously described [27]. qPCR was used to quantify the DNA concentration in the pool employing a 7900HT Fast Real-Time PCR System (Life Technologies, Thermo Fisher Scientific, Carlsbad, CA, USA) and KAPA Library Quantification Kits for Illumina Platform (Kapa Biosystems, Inc., Wilmington, NC, USA). The final pool, at a concentration after dilution between 5 and 20 nM, was used for sequencing as suggested previously [27]. The sequencing steps were performed using a paired-end sequencing run in a MiSeq Illumina device.

### 2.3. 16S rRNA Gene Sequence Analysis

The targeted gene regions were analysed using the FROGS bioinformatics pipeline established by Vaiomer SAS (Labège, France) [28]. The following filters were applied as previously suggested [27]: (1) amplicons with a length < 350 nt or a length > 480 nt were removed; (2) amplicons without the two PCR primers were removed (10% of mismatches were authorised); (3) amplicons with at least one ambiguous nucleotides (‘N’) were removed; (4) operational taxonomic units (OTU) identified as chimera (with search v1.9.5) in all samples in which they were presented were removed; (5) OTU with an abundance lower than 0.005% of the whole dataset abundance were removed, and (6) OTU with a strong similarity (coverage and identity ≥ 80%) with the phiX (library used as a control for Illumina sequencing runs) were removed. OTU were produced via single-linkage clustering, and taxonomic assignment was performed by Blast+ v2.2.30+ with the databank RDP v11.4.

### 2.4. Biochemical Analysis

Routine clinical analyses were collected from our patients’ clinical records, namely, urea, proteinuria, albumin, haemoglobin, cholesterol, low-density lipoprotein (LDL), high-density lipoprotein (HDL), triglycerides, phosphorus (P), calcium (Ca), calcium phosphate product, ferritin, B-type natriuretic peptide (BNP), parathyroid hormone (PTH), sedimentation velocity (SV), CRP, creatinine clearance (Ccreat), residual renal function, and Kt/V (urea). Kt/V (urea) is a parameter that measures adequacy to PD using urea weekly clearance normalised by urea estimated distribution volume. Tumour necrosis factor α (TNF-α), IL-1, IL-6, IL-10 were determined in plasma by Luminex Multiplex Assay (Millipore Corporation, Billerica, MA, USA). ELISA kits were used to evaluate Lipopolysaccharide-binding protein (LPS-BP, Cloud-clone Corp.^®^, Katy, TX, USA), Toll-like receptor 4 (TLR4, Cloud-clone Corp.^®^, Katy, TX, USA), and soluble CD14 (sCD14, Quantikine^®^ ELISA, R&D Systems, Inc., Minneapolis, MN, USA), and TMAO (MyBiosource^®^, San Diego, CA, USA) whereas endotoxins were evaluated by Traditional Kinetic Limulus Amebocyte Lysate (LAL) Assay (Lonza Walkersville, Inc., Walkersville, MA, USA).

Uremic toxins were quantified following the method described by [29] with modifications. p-Cresol sulfate (PCS), 3-indoxyl sulfate (3-INDS), and indole-3-acetic acid (3-IAA) were detected by high-performance liquid chromatography (HPLC) with fluorescence detection (275 and 330 nm). Elution was performed in gradient mode using as mobile phase a mixture of (A) aqueous NaH_2_PO_4_ buffer (20 mM, pH 4.6), containing tetrabutyl ammonium iodide (TBAI, 5 mM), and (B) acetonitrile, at a flow rate of 1.5 mL/min, and injection volume of 20 µL. Prior to HPLC analysis, 100 µL of each plasma standard or sample was added to 300 µL of ethanol containing 0.22 mg/L of internal standard 4-ethylphenol. After vortexing during 30 s, 100 mg of NaCl were added and mixed vigorously. After 10 min, 700 μL of component (A) of mobile phase was further added following centrifugation at 18,000× *g* for 10 min at 4 °C and supernatant analysis by HPLC.

### 2.5. Statistics

All the results are represented as mean ± standard deviation (SD) or in percentage (%). Statistical analysis was performed using SPSS Statistics version 27 (IBM). The categorical variables were described through absolute or relative frequencies (%) and analysed using the Pearson’s chi-square test or Fisher’s exact test when more than 1 cell displayed expected counts less than 5. Continuous variables were described using mean ±SD and analysed by Student’s *t* test for independent samples when following a normal distribution, or by Mann-Whitney U test when there was no normality of the data. Normality was assessed by the Shapiro-Wilk test. A partial correlation between vascular calcification and all-cause mortality risk, while controlling of the effect of age and sex, was performed using JASP-stats software. For all analysis, statistical significance was assumed when *p* values were less than 0.05.

Primer v7 (PRIMER-e, Auckland, New Zealand) was used for the calculation of diversity indices, non-metric multidimensional scaling (NMDS) and principal coordinate analyses (PCO), and other multivariate analyses, mainly ANOSIM and PERMANOVA, were used to test the significance of Beta-diversity. The percentage of OTU data per sample was used for these analyses, followed by squared root transformed data, resemblance matrices of similarity data types using Bray-Curtis similarities, adding dummy value and testing 4999 permutations. The reads in each sample were converted into percentage values according to the total number of sequences in the sample to eliminate the effect of the final number of reads [30]. Post-hoc analyses were done in STAMP 2.1.3 [31] for multiple groups using one-way analysis of variance (ANOVA), Tukey-Kramer (0.95) and Eta-squared for effect size, while, with two groups, analysis using Welch’s *t*-test was conducted (two-sided, Welch’s inverted for confidence interval method).

## 3. Results

Our 44 CKD-PD patients presented an Adragao score mean of 2.98 ± 2.74, included 26.1% patients without VC (Adragao score = 0); 30.4% with moderate VC (Adragao score of 1 or 2) and 39.1% with severe VC (Adragao score higher than 2). In our study, we compared CKD-PD patients with moderate or severe VC versus patients with no VC. Demographic and clinical characteristics of the studied CKD-PD population with and without VC are shown in Table 1.

CKD-PD patients with moderate or severe VC were older and included more males than CKD-PD patients without VC. Concerning the comorbidities, no differences were found in terms of arterial hypertension (present in 95.5% of the studied population), obesity (11.4% of the studied population, with all obese patients presenting VC), or CVD (25.0% of the studied population). A significantly higher prevalence of patients with diabetes mellitus was observed in the group with VC in comparison to the group without VC (43.8% vs. 8.3%, *p* = 0.035).

Most PD technical parameters did not differ significantly between patients with and without VC, except total Kt/V (urea), which was lower in CKD-PD patients with VC (Table 1). In addition, this parameter was inversely correlated with VC severity (Spearman correlation, correlation coefficient = −0.437, *p* < 0.01).

The analysis of the mean values of Charlson Index showed that CKD-PD patients with VC presented a significant increase in all-cause mortality risk compared with CKD-PD patients without VC (5.6 ± 2.2 vs. 3.92 ± 3.0, *p* < 0.05). Accordingly, CKD-PD patients with VC included twice as many patients with severe Charlson Index than patients without VC (Table 1). When VC severity was correlated with all-cause mortality risk, we observed a significant positive correlation (spearman correlation, correlation coefficient (r) = 0.538, *p* < 0.001), meaning that patients with more severe VC present higher mortality risk. Moreover, by multivariable analysis, we found that vascular calcification correlates with the all-cause mortality risk, independently of sex and age.

Pharmacological therapies did not differ significantly between patients with or without VC regarding iron supplementation, erythropoietin, laxatives, hypouricemic agents, statins, calcimimetics, calcium-based phosphate binders, non-calcium-based phosphate binders, and vitamin D. However, the percentage of CKD-PD patients on vitamin D analogues and activators of vitamin D receptor (including alpha D, calcitriol, paricalcitol, and vitamin D receptor selective activators) was 100% in patients without VC whereas it was only ~72% in patients with VC, representing a statistically significant difference (*p* < 0.05). Further, two patients were on chronic anti-inflammatory drugs (prednisolone), both with severe VC (Adragao score of 8), and only three patients were not on anti-hypertensive drugs, all with VC.

Regarding biochemical parameters, only phosphorous plasma levels were significantly lower in CKD-PD patients with VC than patients without VC. Moreover, markers of inflammation (IL-1β, IL-6, TNF-α, and the anti-inflammatory IL-10), markers of intestinal translocation (endotoxins, LPS-binding protein, TLR4, and sCD14), and uremic toxins of microbial origin (T-MAO, PCS, 3-INDS, and 3-IAA) did not differ significantly between patients with or without VC. Regarding sCD14, although no statistically significant differences were found between CKD-PD patients with and without VC, a positive correlation was observed between sCD14 levels and VC severity (r = 0.338, *p* < 0.05). So, CKD-PD patients with more severe VC presented higher plasma values of sCD14.

**Figure 1:**
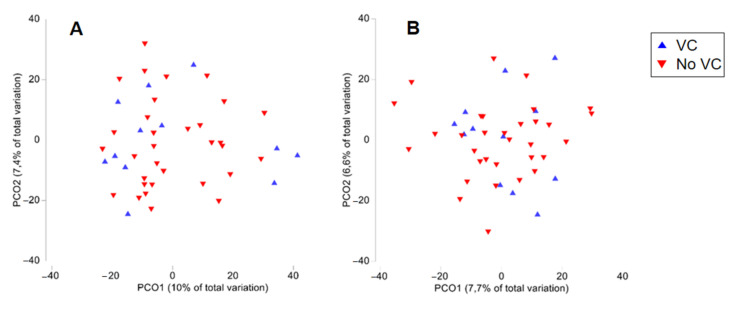
Principal coordinates analysis (PCO) of gut (**A**) and blood (**B**) microbiome in chronic kidney disease patients on peritoneal dialysis with vascular calcification (VC) or without vascular calcification (No VC).

The bacterial microbiome was evaluated in stool samples and whole blood samples. Stool samples displayed a median of 32,370 reads (range: 15,879–41,566). A median of 105 OTUs was observed per sample, with samples presenting between 39 and 216 OTUs. Blood samples displayed a median of 43,131 reads (range: 17,494–50,646). A median of 39 OTUs was observed per sample, with samples presenting between 25 and 56 OTUs. Alpha-diversity analysis was calculated by Shannon index; gut samples showed an average of 4.2 (values ranging from 3.03 to 4.89), while blood samples showed an average of 2.9 (values from 2.3 to 3.3). Similar values of diversity were observed in both groups of patients (with or without VC) separately regarding gut and blood samples. Beta-diversity assessment did not show differences in the gut and blood microbial communities when comparing PD patients with and without VC (Figure 1).

**Figure 2:**
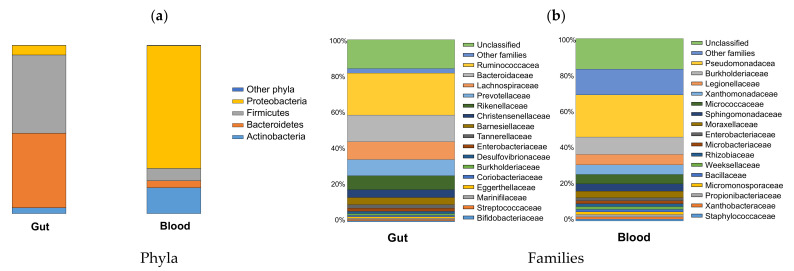
Relative abundance of bacteria phyla (**a**) and family (**b**) in the gut and blood microbiome in chronic kidney disease patients on peritoneal dialysis.

ANOSIM and PERMANOVA confirmed the PCO observations, as the groups for both analyses were not significantly different (*p* > 0.1). Therefore, the taxonomic profiles of the gut and blood microbiome were similar at phylum and family taxonomic levels within each group of patients with or without VC (Figure 2).

**Figure 3:**
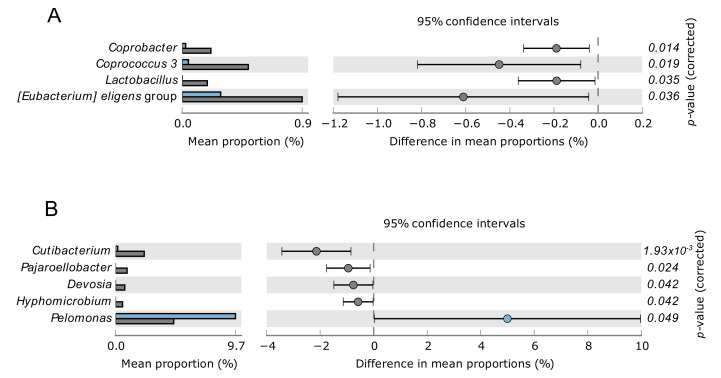
Relative changes of gut (**A**) and blood (**B**) bacterial taxa at the genus/family level in chronic kidney disease patients on peritoneal dialysis comparing patients with vascular calcification (grey bars) with patients without vascular calcification (blue bars).

Gut microbiome was dominated by Firmicutes and Bacteroidetes at the phylum level, and by Ruminococcaceae, Bacteroidaceae, Lachnospiraceae, and Prevotellaceae at family level. The blood microbiome was dominated by Proteobacteria and Actinobacteria at the phylum level, and by Pseudomonadaceae, Burkholderiaceae, and Legionellaceae at family level. Nonetheless, relative changes of specific rare and/or less abundant taxa were observed between CKD-PD patients with and without VC, namely *Coprobacter*, *Coprococcus 3*, *Lactobacillus*, and *Eubacterium eligens* group in gut microbiome, and *Cutibacterium*, *Pajaroellobacter*, *Devosia*, *Hyphomicrobium*, and *Pelomonas* in blood microbiome (Figure 3).

**Figure 4:**
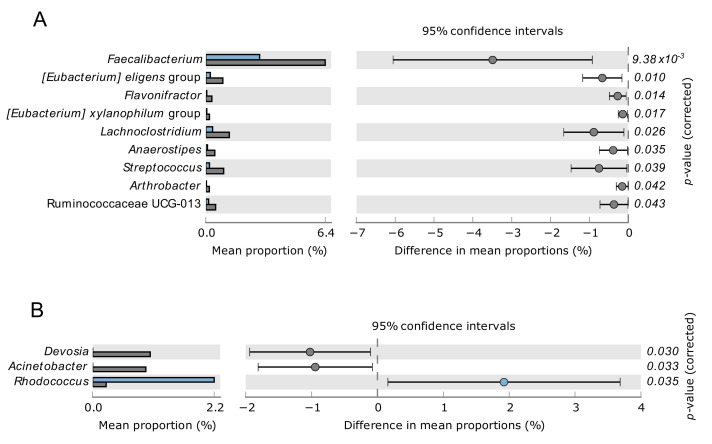
Relative changes of gut (**A**) or blood (**B**) bacterial taxa at the genus/family level in chronic kidney disease patients on peritoneal dialysis comparing patients with low all-cause mortality risk (Charlson Index scores of 2 or less, blue bars) with patients with moderate or severe all-cause mortality risk (Charlson Index scores of 3 or more, grey bars).

Given the correlation between VC and all-cause mortality risk, we explored the gut and blood microbiome differences between CKD-PD patients with low and high mortality risk (Figure 4).

Among the taxonomic differences observed in CKD-PD patients with and without VC, patients with high mortality risk presented higher relative abundance in *E. eligens* group in the gut microbiome and *Devosia* in the blood microbiome when compared to patients with low mortality risk.

Given that patients with VC included more male and older participants, we further investigate if sex and age would play a role in the relative changes of gut or blood microbiome (Figures S1 and S2). We found that male participants also have higher levels of *E. eligens* group in the gut in comparison to females. Although *Hyphomicrobium* was elevated in patients with VC in comparison to patients without VC, we found that *Hyphomicrobium* was present in adult participants but not in senior participants. Therefore, except for *E. eligens* group, the results suggest that the variation of the specific taxa in Figure 3 and Figure 4 are mostly explained by vascular calcification in CKD-PD patients.

## 4. Discussion

Our results showed relative changes in specific taxa between CKD-PD patients with and without VC, namely *Coprobacter*, *Coprococcus 3*, *Lactobacillus*, and *E. eligens* group in the gut, and *Cutibacterium*, *Pajaroellobacter*, *Devosia*, *Hyphomicrobium*, and *Pelomonas* in the blood. An association between VC and all-cause mortality risk in CKD-PD patients was also observed, and patients with higher mortality risk corroborate the changes of *E. eligen*s in the gut and *Devosia* genus in the blood. Although we did not find differences in uremic toxins, intestinal translocation markers, and inflammatory parameters among CKD-PD patients with and without VC, sCD14, a nonspecific marker of monocyte activation, was positively correlated with VC severity, suggesting its association with inflammation. Collectively, these results open new avenues for biomarkers discovery in CKD-PD patients.

The gut microbiome of our CKD-PD population was dominated by Firmicutes and Bacteroidetes at the phylum level, as described in healthy individuals, and by Ruminococcaceae, Bacteroidaceae, Lachnospiraceae, and Prevotellaceae at the family level, following other studies describing the gut microbiome of CKD-PD patients [32,33,34]. Despite only a few taxa differed between CKD-PD patients with and without VC, these taxa represent relevant groups among the gut microbiome, such as Coprobacter, *Coprococcus*, *Lactobacillus* or *Eubacterium*, which were more abundant in CKD-PD patients with VC. Some of these taxa are key players in the gut microbiome [35,36,37,38] and may be altered when the gut microbiome becomes dysbiotic, for example, in CKD patients [11]. Among the taxonomic differences observed in the gut microbiome for CKD-PD patients with or without VC, patients with higher mortality risk also demonstrated higher relative abundance in *E. eligens* group, highlighting a potential critical role of this taxon in CKD-PD patients. However, the microbiome differences associated to the sex may have contributed to this result, given that participants with VC include more males, and male participants also presented higher *E. eligens* group prevalence in comparison to females. The increase in the relative abundance of *E. eligens* group is most frequently associated with a healthy status [39,40,41]. For example, *E. eligens* were depleted in stool samples from atherosclerotic patients from Sweden and China cohorts and were appointed as promising probiotics and potential therapeutic targets for atherosclerosis [40]. However, the relative abundance of *E. eligens* group in the gut has also been found, occasionally, associated with disease [42]. Taking our results into account, the increase in *E. eligens* may not always constitute a protective factor as has been reported in previous studies.

Although still controversial, there is evidence supporting the existence of a healthy non-infectious human blood-microbiome [15,17,43]. In our CKD-PD patients, the blood microbiome was dominated by Proteobacteria and Actinobacteria at the phylum level and by Pseudomonadaceae, Burkholderiaceae, and Legionellaceae at the family level. Similarly, Shah et al. [20] observed that Pseudomonadaceae and Enterobacteriaceae families were significantly higher in the blood microbiome of non-dialysis CKD patients than in healthy controls. They demonstrated higher Proteobacteria and Actinobacteria predominance in the blood in contrast to Bacteroidetes and Firmicutes predominance in the gut. Proteobacteria is a major phylum of Gram-negative bacteria, which includes a wide variety of pathogens such as *Escherichia*, *Salmonella*, *Vibrio*, *Yersinia*, *Pseudomonas*, *Burkholderia*, *Legionella*, and many other genera. Proteobacteria are higher both in the gut and blood in many chronic inflammatory diseases, including inflammatory bowel disease, metabolic syndrome, cardiovascular diseases, and chronic lung diseases. They have also been detected in atherosclerotic plaques and been related to the progression of CKD [17,20,44]. The correlation of all these diseases with gut dysbiosis, intestinal bacterial translocation, and endotoxaemia-related inflammation, as well as the clinical association between one and the other, suggests a common mechanism underlying these diseases associated with inflammation arising from the gut.

It is also relevant to note that families found in the blood microbiome of our CKD-PD patients include serious clinical pathogens, such as Pseudomonadaceae, Burkholderiaceae, and Legionellaceae. When evaluating the infection history of these patients, five presented previous *Pseudomonas aeruginosa* infections (between 4 months to 2 years before), with this pathogen being isolated from the catheter exit-site in four of these five patients, and in the respiratory tract in the remaining patient. However, it is important to highlight that the blood microbiome was evaluated through the detection of short sequences of bacterial genetic material, specifically the V3–V4 variable regions of the 16S rRNA gene. Therefore, these genetic sequences may result from circulating microbial DNA derived from phagocyted microbial cells of microorganisms translocated from the gut, the oral cavity, the PD catheter biofilm, or even from PD solutions [18,45,46]. Notwithstanding, the hypothesis that some of these DNA sequences may originate from living microbes should not be discarded, given the fact that viable bacteria have been found in blood from donors reported as medically healthy [47].

We observed in the blood microbiome of CKD-PD patients with VC an increase in *Cutibacterium*, *Pajaroellobacter*, *Devosia*, and *Hyphomicrobium*, and a decrease in relative abundance of *Pelomonas* when compared to CKD-PD patients without VC. Most of these groups appear sporadically in different areas of the human microbiome (skin, oral, gut) [48,49,50,51], but the real role of these genera remains unknown. An increase in the relative abundance of *Devosia* genus was found both in CKD-PD patients with VC when compared with CKD-PD patients without VC, as well as in CKD-PD patients with a higher mortality risk. To our knowledge, *Devosia* has not been reported previously in the blood microbiome but has been found to be increased in the gut microbiota of colorectal cancer patients [51] and in rabbits with heat stress [52], suggesting its possible translocation from the gut into the systemic circulation.

In our work, we also measured markers of intestinal translocation (endotoxins, LPS-binding protein, TLR4, and sCD14), inflammatory parameters (C-reactive protein, ferritin, sedimentation velocity, IL-1β, IL-6, TNF-α, and the anti-inflammatory IL-10), uremic toxins (PCS, 3-INDS, 3-IAA, and TMAO), and other routine laboratory parameters (such as urea, proteinuria, albumin, haemoglobin, cholesterol and its different fractions, triglycerides, calcium, parathormone, BNP), but no statistical significant differences were found between CKD-PD patients with or without VC. Although markers of intestinal translocation, uremic toxins, or inflammatory parameters are known to be increased in CKD patients [53,54,55], it should be noted that our study population included only end-stage kidney disease patients, and not healthy controls for comparison. The absence of differences between CKD-PD patients with or without VC could be associated with the relatively small number of patients included in this study.

Interestingly, we found that sCD14, a human monocyte differentiation antigen that acts as a pattern recognition receptor and is a TLR co-receptor for the detection of pathogen-associated molecular patterns such as lipopolysaccharides [56], was positively correlated with VC severity. In accordance, plasma sCD14 levels have been independently associated with myocardial infarction, coronary heart disease, and all-cause mortality among men and women above 65 years old in the Cardiovascular Health Study [57]. Longenecker et al. [22] observed that sCD14 was independently associated with coronary artery calcification measured by computed tomography and also predicted the extent of subclinical disease in other vascular beds in HIV patients. Poesen et al. [58] demonstrated that sCD14 was elevated in patients with decreased kidney function and was associated with mortality and CVD in patients with CKD not yet on dialysis during a median follow-up of 52–54 months. Other studies positively related higher levels of sCD14 level to markers of inflammation and negatively to nutritional status and concluded sCD14 to be an independent predictor of all-cause mortality in long-term haemodialysis patients [59,60]. Together, these findings support a putative role of sCD14 in VC that should be explored in future studies in CKD-PD population.

When comparing CKD-PD patients with and without VC we observed higher estimated mortality risk in patients with VC, corroborating previous reports [61]. In our study, the CKD-PD patients with VC included more males, older patients, and a higher prevalence of diabetes in comparison with CKD-PD patients without VC. In fact, these three factors were previously recognised as major contributors to VC [62,63]. Moreover, we also observed lower Kt/V (urea) values in CKD-PD patients with VC when compared with patients without VC. In accordance, lower Kt/V values have been associated with VC and CVD in dialysis patients, including PD and haemodialysis patients [61,64].

When comparing phosphorous levels between CKD-PD patients with or without VC, we unexpectedly found higher phosphorous levels in patients without VC. We also found higher levels of calcium-phosphate product in patients without VC when compared with patients with VC but below the cut-off established for higher risk of VC and CVD in end-stage CKD patients [65]. According to KDIGO guidelines [66] and previously published articles [67,68], VC is marked by hyperphosphataemia and higher levels of calcium-phosphate product. Perhaps our results could be explained by some peculiarities in our study population. We performed a unique blood test and we did not collect samples in different time-points, so it is possible our CKD-PD patients with VC presented higher phosphorous levels in the past. Another argument is that PD patients on vitamin D analogues and activators of vitamin D receptor (including alpha D, calcitriol, paricalcitol, and vitamin D receptor selective activators) represented 100% of patients without VC, and only ~72% of patients with VC, denoting a significant difference (*p* < 0.05). The relationship between vitamin D and VC is complex. Moderate activation of vitamin D receptor (VDR) signaling protects against VC, but a deficient or excessive activation of VDR has been associated to VC [69]. As some studies proved that clinically relevant dosages of calcitriol and paricalcitol may protect against VC [70], others found no differences in the presence of VC in PD patients treated with calcitriol or calcium-based phosphate binders [71]. Vitamin D analogues and activators of vitamin D receptor promote an increase in phosphate levels through different mechanisms, so the higher intake of these drugs in the group without VC may collaborate on the higher levels of phosphorous in that group. Another argument to be looked at with caution is that, although not statistically significant, in our study we observed in the group with VC a higher calcium-based phosphate binders intake and lower non-calcium-based phosphate binders intake, resulting in better phosphorous control in that group. In clinical trials for the pharmacological management of phosphate imbalance, phosphate binders, especially non-calcium-based phosphate binders, were reported to low serum phosphorous levels by decreasing fibroblast growth factor-23 (FGF-23), which has been shown to stimulate phosphorous excretion and reduce VC [68] with protective effects on VC [72].

Lastly, it would be more accurate to evaluate VC using coronary computed tomography instead of Adragao score; however, the simplicity of the used method is of great advantage in clinical studies [24]. Adragao score measures VC and therefore may estimate CVD risk in CKD patients through hands and pelvic radiographies. Charlson Comorbidity Index predicts 10-year survival in patients with multiple comorbidities and has been useful in the prediction of mortality risk in CKD patients [73].

## 5. Conclusions

Vascular calcification is a highly frequent condition in CKD and a well-established risk factor for the development of CVD in CKD patients. Traditional factors fall short in explaining the high prevalence of VC and CVD in kidney disease, suggesting the involvement of a CKD-specific pathological pathway that remains unknown. In recent years, gut dysbiosis has been shown to contribute to CVD, inflammation, and VC in CKD patients, but nothing was so far known regarding the role of gut microbiome in CKD-associated VC and CVD. Moreover, the information regarding blood microbiome and its putative relevance in health and disease is still very scarce.

**Figure 5:**
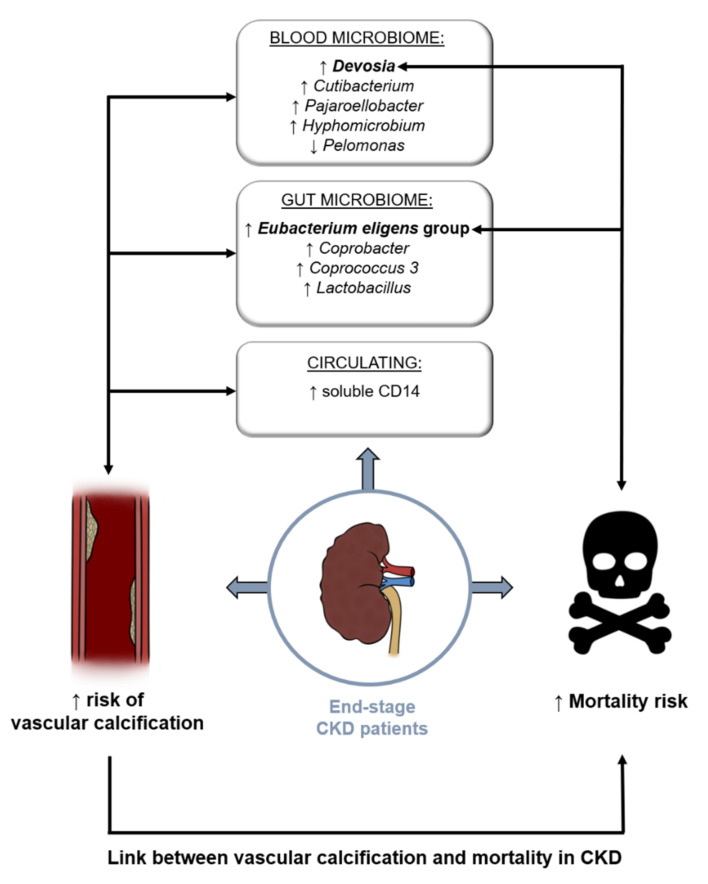
Our results suggest that specific taxa in the gut microbiome (*Coprobacter*, *Coprococcus 3*, *Lactobacillus*, and *Eubacterium eligens* group) and in the blood microbiome (*Cutibacterium*, *Pajaroellobacter*, *Devosia*, *Hyphomicrobium*, and *Pelomonas*) are different between CKD-PD patients with and without VC. sCD14 (a nonspecific marker of monocyte activation) correlated with vascular calcification (VC) severity in CKD-PD patients. An association between VC and all-cause mortality risk in CKD-PD patients was observed and patients with higher mortality risk corroborate the changes of *Eubacterium eligen*s in the gut and *Devosia* genus in the blood.

Our results showed relative changes of specific taxa between CKD-PD patients with and without VC, namely regarding *Coprobacter*, *Coprococcus 3*, *Lactobacillus*, and *E. eligens* group in gut, and *Cutibacterium*, *Pajaroellobacter*, *Devosia*, *Hyphomicrobium*, and *Pelomonas* in the blood. Relative changes in the *E. eligens* group may also be associated with higher male prevalence in the group of participants with vascular calcification. An association between VC and all-cause mortality risk in CKD-PD patients was also observed, and patients with higher mortality risk corroborated the changes of *E. eligen*s in the gut and *Devosia* genus in the blood. Although we did not find differences in uremic toxins, intestinal translocation markers, and inflammatory parameters among CKD-PD patients with and without VC, sCD14, a nonspecific marker of monocyte activation, was positively correlated with VC severity, suggesting an association with low grade inflammation. Figure 5 shows a schematic view of our results.

In conclusion, our results suggest a role as biomarkers of gut *E. eligens* group, blood *Devosia*, and circulating sCD14 in CKD-VC, CVD, and mortality risk that should be further explored.

## Figures and Tables

**Table 1:** Demographic and clinical characterization of chronic kidney disease patients on peritoneal dialysis (CKD-PD) with and without vascular calcification (VC).

	CKD-PD (*n* = 44)	CKD-PD With no VC (*n* = 12)	CKD-PD with VC (*n* = 32)	*p*-Value
**Demographic data**				
Age, years	56.1 ± 10.9	47.7 ± 11.5	59.4 ± 8.8	**<0.001** ^a^
Sex, % male	65.9%	33.3%	78.1%	**0.011** ^d^
**PD parameters**				
PD duration, months	33.4 ± 30.0	36.3 ± 43.4	30.9 ± 23.8	0.668 ^b^
PD type, %				>0.999 ^d^
APD	52.3%	50.0%	53.1%	
CAPD	47.7%	50.0%	46.9%	
Ccreat, L/week	114.8 ± 56.8	105.7 ± 45.1	118.2 ± 60.8	0.668 ^b^
Residual renal function, mL/min	5.6 ± 4.0	5.8 ± 3.8	5.6 ± 4.1	0.706 ^b^
Kt/V (urea)	2.2 ± 0.5	2.6 ± 0.6	2.1 ± 0.4	**0.004** ^b^
Charlson Index, %				**0.003** ^c^
Low (≤2)	18.2%	50.0%	6.3%	
Moderate (3–4)	31.8%	25.0%	34.4%	
Severe (≥5)	50.0%	25.0%	59.4%	
**Biochemical parameters**				
Urea, mg/dL	125.0 ± 37.0	127.6 ± 20.1	124.0 ± 41.8	0.780 ^a^
Proteinuria mg/24 h	1.0 ± 1.2	0.9 ± 1.0	1.0 ± 1.2	0.342 ^b^
Albumin, g/L	37.1 ± 3.3	37.0 ± 2.6	37.1 ± 3.6	0.944 ^a^
Hemoglobin, g/dL	11.5 ± 1.4	11.0 ± 0.9	11.7 ± 1.6	0.133 ^a^
Cholesterol, mg/dL	171.0 ± 56.8	169.9 ± 42.8	171.4 ± 61.8	0.825 ^b^
LDL, mg/dL	95.7 ± 42.6	99.9 ± 33.7	94.0 ± 46.1	0.547 ^b^
HDL, mg/dL	45.6 ± 10.7	47.4 ± 9.3	45.0 ± 11.3	0.267 ^b^
Triglycerides, mg/dL	158.6 ± 68.4	129.8 ± 42.9	169.4 ± 73.5	0.169 ^b^
P, mg/dL	5.0 ± 1.1	5.72 ± 1.05	4.73 ± 1.02	**0.011** ^a^
Ca, mg/dL	9.02 ± 0.89	9.39 ± 0.85	8.84 ± 0.89	0.073 ^a^
Ca • P product	43.83 ± 10.63	52.08 ± 9.32	40.67 ± 9.70	**0.002** ^b^
Ferritin, ng/mL	361.3 ± 222.9	316.1 ± 221.3	378.3 ± 224.6	0.419 ^a^
BNP, pg/mL	143.1 ± 119.2	87.0 ± 36.6	163.1 ± 131.9	0.124 ^b^
PTH, pg/mL	462.5 ± 280.0	485.5 ± 366.4	453.9 ± 246.7	0.866 ^b^
SV, mm	64.2 ± 25.6	67.2 ± 18.7	63.1 ± 27.9	0.644 ^a^
CRP, mg/L	5.3 ± 8.5	4.8 ± 7.7	5.5 ± 8.9	0.907 ^b^
TNF-α, pg/mL	11.4 ± 4.3	10.4 ± 2.8	11.7 ± 4.7	0.524 ^b^
IL-1β, pg/mL	1.3 ± 0.93	1.3 ± 1.0	1.3 ± 0.9	0.969 ^b^
IL-10, pg/mL	17.7 ± 14.7	17.5 ± 16.7	17.8 ± 14.2	0.825 ^b^
IL-6, pg/mL	2.9 ± 6.3	5.4 ± 10.3	2.0 ± 3.8	0.687 ^b^
Endotoxins, EU/mL	3.8 ± 0.8	3.8 ± 0.4	3.7 ± 0.8	0.978 ^a^
LPS-BP, µg/mL	39.9 ± 17.1	32.2 ± 13.4	41.2 ± 18.3	0.442 ^b^
TLR-4, pg/mL	624.4 ± 439.2	699.1 ± 464.5	596.4 ± 433.7	0.630 ^b^
sCD14, µg/mL	5.0 ± 2.1	4.4 ± 2.0	5.3 ± 2.1	0.224 ^b^
T-MAO	0.52 ± 0.62	0.47 ± 0.40	0.57 ± 0.70	0.854 ^b^
PCS, mg/L	33.5 ± 19.1	36.4 ± 18.0	32.3 ± 19.7	0.341 ^b^
3-INDS, mg/L	23.7 ± 14.6	24.1 ± 9.6	23.5 ± 16.22	0.442 ^b^
3-IAA, mg/L	1.1 ± 1.2	1.0 ± 0.5	1.1 ± 1.4	0.169 ^b^

Results are shown in absolute or relative frequencies (%) or mean ± standard deviation (SD). CKD, chronic kidney disease; PD, peritoneal dialysis; APD, Automated Peritoneal Dialysis; CAPD, continuous ambulatory peritoneal dialysis; Ccreat, creatinine clearance; residual renal function; Kt/V (urea); LDL, low-density lipoprotein; HDL, High-density lipoprotein; P, phosphorous; Ca, calcium; Ca·P product, calcium phosphate product, BNP, B-type natriuretic peptide; PTH, Parathyroid hormone; SV, sedimentation velocity, CRP, C reactive protein; TNF-α, Tumour necrosis factor-α; IL, Interleukin; LPS-BP, Lipopolysaccharide-binding protein; TLR-4, Toll-like receptor 4; sCD14, soluble CD14; TMAO, trimethylamine N-oxide; PCS, p-cresol sulphate; 3-INDS, 3-indoxyl sulfate; 3-IAA, indole-3-acetic acid. *p* values were calculated using the following statistical analysis: ^a^ Student’s *t*-test, ^b^ Mann-Whitney U test, ^c^ Pearson Chi-square test, and ^d^ Fisher test.

## Data Availability

The data supporting reported results can be found at NCBI under BioProject: PRJNA819486.

## Supplementary Materials

The following supporting information can be downloaded at: https://www.mdpi.com/article/10.3390/biom12070867/s1. Figure S1: Relative changes of gut (**A**) or blood (**B**) bacterial taxa at the genus/family level in chronic kidney disease patients on peritoneal dialysis comparing male (yellow bars) with female (blue bars) patients. Figure S2: Relative changes of gut (**A**) or blood (**B**) bacterial taxa at the genus/family level in chronic kidney disease patients on peritoneal dialysis comparing adulthood (until 65 years old, grey bars) with seniorhood (>65 years old, green bars) patients.
