# Supplementary material for: Vascular Calcification and the Gut and Blood Microbiome in Chronic Kidney Disease Patients on Peritoneal Dialysis: A Pilot Study
Source: Biomolecules. 2022 Jun 21;12(7):867. doi: 10.3390/biom12070867 (PMC9313079; doi:10.3390/biom12070867)
Supplement: Supplementary file 1 [file biomolecules-12-00867-s001.zip › biomolecules-1715322-supplementary.pdf]

Supplementary Material

Vascular Calcification and the Gut and Blood Microbiome in Chronic Kidney Disease Patients on Peritoneal Dialysis: A Pilot Study

Ana Merino-Ribas [1,2,3], Ricardo Araujo [1], Luciano Pereira [1,4], Joana Campos [1], Luísa Barreiros [5], Marcela A. Segundo [5], Nádia Silva [4], Carolina F. F. A. Costa [1,6], Janete Quelhas-Santos [7], Fábio Trindade [7], Inês Falcão-Pires [7], Ines Alencastre [1], Ioana Bancu Dumitrescu [2,8] and Benedita Sampaio-Maia [1,9,*]

[1] Nephrology & Infectious Diseases R & D Group, i3S—Instituto de Investigação e Inovação em Saúde, INEB—Instituto de Engenharia Biomédica, Universidade do Porto, 4200-135 Porto, Portugal; anamerinoribas@gmail.com (A.M.-R.); ricardo.araujo@i3s.up.pt (R.A.); lucianoarturpereira@hotmail.com (L.P.); joanaaraujocampos@gmail.com (J.C.); carolina.costa@i3s.up.pt (C.F.F.A.C.); ines.alencastre@ineb.up.pt (I.A.)

[2] Departament de Medicina, Universitat Autonoma de Barcelona, 08035 Barcelona, Spain; ioana_bancu@yahoo.com

[3] Nephrology Department, Hospital Universitari de Girona Doctor Josep Trueta, 17007 Girona, Spain

[4] Nephrology Department, Centro Hospitalar Universitário de São João, 4200-319 Porto, Portugal; nadiaraquel77@gmail.com

[5] LAQV, REQUIMTE, Departamento de Ciências Químicas, Faculdade de Farmácia, Universidade do Porto, 4050-313 Porto, Portugal; lbarreiros78@gmail.com (L.B.); msegundo@ff.up.pt (M.A.S.)

[6] Instituto de Ciências Biomédicas Abel Salazar, Universidade do Porto, 4050-313 Porto, Portugal

[7] UnIC@RISE- Cardiovascular Research and Development Centre, Department of Surgery and Physiology, Faculty of Medicine, University of Porto, 4200-319 Porto, Portugal; sjanete@med.up.pt (J.Q.-S.); ftrindade@med.up.pt (F.T.); ipires@med.up.pt (I.F.-P.)

[8] Fresenius Nephrocare, 110372 Pitesti, Romania

[9] Faculdade de Medicina Dentária, Universidade do Porto, 4200-393 Porto, Portugal

* Correspondence: bmaia@fmd.up.pt; Tel.: +351-220-901-100

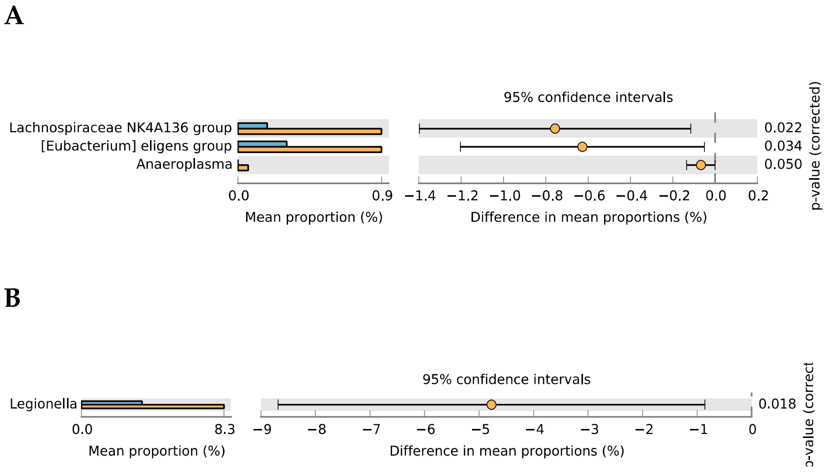

Figure S1. Relative changes of gut **(A)** or blood **(B)** bacterial taxa at the genus/family level in chronic kidney disease patients on peritoneal dialysis comparing male (yellow bars) with female (blue bars) patients.

A

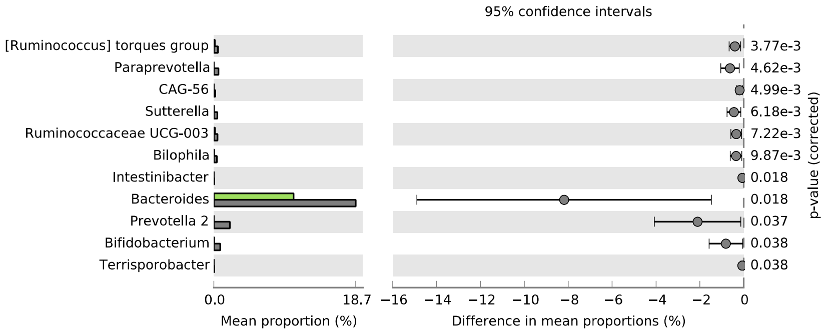

B

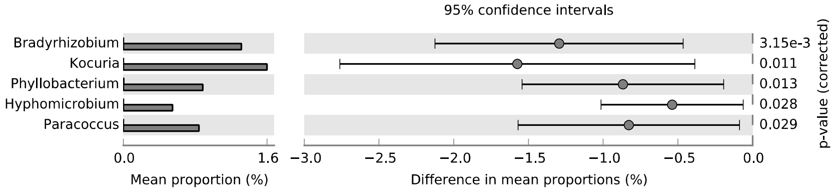

Figure S2. Relative changes of gut **(A)** or blood **(B)** bacterial taxa at the genus/family level in chronic kidney disease patients on peritoneal dialysis comparing adulthood (until 65 years old, grey bars) with seniorhood (> 65 years old, green bars) patients.
